# The Hepatoprotective Effect of Taurisolo, a Nutraceutical Enriched in Resveratrol and Polyphenols, Involves Activation of Mitochondrial Metabolism in Mice Liver

**DOI:** 10.3390/antiox9050410

**Published:** 2020-05-11

**Authors:** Nadia Badolati, Raffaello Masselli, Eduardo Sommella, Serena Sagliocchi, Alessandro Di Minno, Emanuela Salviati, Pietro Campiglia, Monica Dentice, Gian Carlo Tenore, Mariano Stornaiuolo, Ettore Novellino

**Affiliations:** 1 Department of Pharmacy, University of Naples Federico II, Via Montesano 49, 80131 Naples, Italy; nadia.badolati@unina.it (N.B.); raffaello.masselli@unina.it (R.M.); alessandro.diminno@unina.it (A.D.M.); giancarlo.tenore@unina.it (G.C.T.); ettore.novellino@unina.it (E.N.); 2 Department of Pharmacy, School of Pharmacy, University of Salerno, Via Giovanni Paolo II 132, I-84084 Fisciano, SA, Italy; esommella@unisa.it (E.S.); esalviati@unisa.it (E.S.); pcampiglia@unisa.it (P.C.); 3 Department of Clinical Medicine and Surgery, University of Naples Federico II, Via Pansini 5, 80149 Naples, Italy; serena.sagliocchi@unina.it (S.S.); monica.dentice@unina.it (M.D.); 4 PhD Program in Drug Discovery and Development, University of Salerno, I-84084 Fisciano, SA, Italy

**Keywords:** antioxidants, Resveratrol, polyphenols, nutraceuticals, mitochondria, liver

## Abstract

Liver diseases affect millions of people worldwide. In most of the cases, severe hepatic dysfunction and liver cancer stem from mild and common clinical signs including hepatic steatosis, insulin resistance, liver inflammation, and oxidative stress, all together referred to as Nonalcoholic Fatty Liver Disease (NAFLD). Nutraceuticals endowed with antioxidant activity have been shown to reduce NAFLD risk factors and exert hepatoprotective effects. Here, we test the protective effect exerted on liver by the antioxidant Taurisolo, a nutraceutical formulation produced by grape pomace and enriched in Resveratrol and Polyphenols. We analyze the effect of Taurisolo on liver cells by profiling the metabolome of in vitro cultured hepatic HuH7 cells and of C57BL-6J mice fed a High Fat Diet and treated with the nutraceutical. Both in vitro and in vivo, we provide evidence that Taurisolo reduces risk factor markers associated with NAFLD. Taurisolo stimulates glucose uptake and reduces hepatic cholesterol and serum triglycerides. Furthermore, we give new insights into the mechanism of action of Taurisolo. The nutraceutical increases mitochondrial activity and promotes respiration and ATP production, fostering catabolic reactions like fatty acid β-oxidation and amino acid catabolism. On the contrary, Taurisolo reduces anabolic reactions like biosynthesis of cholesterol, bile acids, and plasma membrane lipids.

## 1. Introduction

With a global prevalence of 25%, Nonalcoholic Fatty Liver Disease (NAFLD) is a major cause of liver disease worldwide [1]. Clinical signs of NAFLD include hepatic steatosis, triglyceride (TG) accumulation, insulin resistance (IR), liver inflammation, and oxidative stress, all together leading to a severe condition of hepatic dysfunction and cirrhosis [2,3]. Subjects affected by NAFLD are more prone to develop hepatocellular carcinoma, suggesting a clear correlation between cancer and liver diseases [4,5].

While excessive fat consumption remains the leading cause of NAFLD, dysmetabolism, hypertriglyceridemia, and IR are emerging risk factors for liver dysfunction [6]. Indeed, the syndrome is associated with obesity (51% of co-occurrence), type 2 diabetes (22%), hyperlipidemia (69%), hypertension (39%), and metabolic syndrome (42%) [7,8,9].

Even if lifestyle modification, physical activity, and reduction of caloric intake are established strategies to reduce the occurrence of NAFLD [10,11], no standardized approach is available so far. Due to the high prevalence of the disease, efforts have been made to control risk factors for the NAFLD to ultimately avoid the progression of the condition. As a consequence, drugs administered to prevent hypertriglyceridemia (fibrates, omega-3-fatty acids), hypercholesterolemia (statins, ezetimibe, Proprotein convertase subtilisin/kexin type 9 (PCSK9) inhibitors, lomitapide, and bile acid sequestrants) [12] and IR (metformin, sulfonylureas and thiazolidinediones, dipeptidyl peptidase-4 inhibitors and incretins) [13] have all shown to ameliorate hepatic dysfunctions caused by NAFLD [3,14].

Several studies reported oxidative stress playing a pivotal role in NAFLD [15], suggesting that antioxidants could be alternative therapeutic strategies to prevent it [14,16]. Nutraceuticals, supplements, and functional foods endowed with antioxidant activity (e.g., Resveratrol, Ursodeoxycholic acid, Vitamin E, Silymarin, Betaine) have been shown to possess hepatoprotective functions, triglyceride- and cholesterol-lowering properties and act as mild antidiabetic agents [16,17]. Pure Resveratrol supplementation has been shown to reduce the risk of obesity and NAFLD [18,19,20]. The beneficial effects of Resveratrol seem to depend on its ability to ameliorate gut microbiota composition and improve gut barrier integrity [19,21,22].

In the last two decades, meta-analyses are documenting diets enriched in antioxidants as capable of reducing NAFLD progression and prevent hepatic irreversible dysfunctions. Due to their high content of Resveratrol [23] and polyphenols [24,25], grapes, grape juice, and grape pomace extracts have been under investigation as alternative, non-pharmacological antioxidants [24,26]. Grape pomace extracts combine the nutraceutical properties of their bioactive fraction to the bio-sustainability of being produced from agricultural and alimentary waste. In the last few years, our group has extensively investigated the antioxidant properties of a grape pomace extract from the grape *cultivar* “Aglianico”. The extract is enriched in Resveratrol, Catechins, and Procyanidins and one of its nutraceutical formulations, Taurisolo, has been included in several over-the-counter nutraceutical products. Administered to humans, Taurisolo acts as an antioxidant and reduces oxidized-LDL serum levels, as a circulating oxidative stress biomarker, and of Trimethylamine N-oxide (TMAO) a known cardiovascular risk factor marker [27,28]. In murine models, both acute and chronic consumption of Taurisolo (i) protects the blood–brain barrier and reduces brain damages in rat undergoing ischemic injuries, (ii) reduces Radical Oxygen Species (ROS) produced by endothelial cells during oxidative stress, (iii) reduces Thromboxane TxB2 biosynthesis and (iv) promotes Nitric Oxide production [28,29].

While the antioxidant activity of Taurisolo would suggest its protective effect on liver cells, its hepatoprotective potential has not yet been verified. Here we start analyzing the effect exerted by Taurisolo on cultured hepatic HuH7 cells and on C57BL-6J mice fed a High Fat Diet (HFD). Our results show that Taurisolo promotes glucose uptake, hepatic glycolysis, fatty acid beta-oxidation leading to an overall reduction of hepatic triglycerides and cholesterol levels and a better response to insulin. We further use a Metabolomic approach to identify the molecular mechanism exerted by Taurisolo on hepatic cells. Both in vitro and in vivo, the nutraceutical acts as a mitochondrial booster and stimulates catabolic reactions of oxidative phosphorylation (OXPHOS) and mitochondrial ATP production [30]. Because of this increased catabolic activity, hepatic cells consume lipids ultimately reducing circulating Triglycerides (TG) [31,32].

## 2. Materials and Methods

### 2.1. Materials

PBS (A0965-9010), CaCl_2_ (A3779-1000), TritonX-100(A1388-0500) were all from Applichem (Darmstadt. Germany). Insulin (I6634), Glycine (50046), Methanol (322415), (2-(*N*-(7-Nitrobenz-2-oxa-1,3-diazol-4-yl)Amino)-2-Deoxyglucose)(2-NBDG, 72987), acetonitrile (34851), 2-Deoxyglucose(2-DG;D6134), (4′,6-diamidino-2-phenylindole) (DAPI, D8417) and BSA(A2153) were from Sigma Aldrich (Taufkirchen, Germany). Formaldehyde (7040) was from JTBaker (Deventer, The Netherlands). Ly294002 was from Tocris (Bristol, UK). MitoTracker Red CMXRos (M7512, Invitrogen, Carlsbad, CA, USA) used for staining of mitochondria was reconstituted in DMSO and 1 mM stock aliquots were stored at −20 °C before use.

### 2.2. Nutraceuticals

Taurisolo was obtained from Aglianico cultivar grape pomace, collected during the harvest in Campania (an administrative region of Italy located on the south-western portion of the Italian Peninsula) during autumn 2016. Large scale production of Taurisolo has been accomplished by MBMed Company (Turin, Italy). The grape pomace was extracted with water at 50 °C. After centrifugation, the extract underwent a spray-drying process with maltodextrins as support, obtaining a fine powder, containing a pomace: maltodextrins ratio of 1.0 ± 0.1 (*w/w*).

High Performance Liquid Chromatography-diode-array detector (HPLC-DAD, Jasco Extrema LC-4000 system (Jasco Inc., Easton, PA, USA)) analysis indicates that the main polyphenols contained in Taurisolo are (values are expressed in μg/g Taurisolo ± standard deviation of three repetitions): Gallic acid 1463.4 ± 65.5; Syringic acid 539.2 ± 6.02, Caffeic acid 20.7 ± 0.76, *p*-coumaric acid 27.9 ± 0.66, Ferulic acid 10.5 ± 0.70, Resveratrol 13.6 ± 0.64, Catechin 4087.0 ± 64.5, Epicatechin 886.0 ± 7.82, Quercetin 40.22 ± 7.11, Rutin 28.4 ± 0.70, Procyanidin B1 dimer 62.8 ± 0.59, Procyanidin B2 dimer 426.5 ± 5.92, Procyanidin B3 dimer 22.05 ± 6.61, Procyanidin B4 dimer 56.6 ± 0.88, Procyanidin C2 trimer 44.6 ± 0.66. Taurisolo is highly soluble in water as well as in PBS, that were used as a vehicle for in vivo and in vitro experiments, respectively. Mother stocks of Taurisolo 40 g/L were freshly prepared in water and filtered with a 0.22 μm Whatman filtering unit to be then diluted in culture medium (in vitro experiments) or in drinking water (in vivo experiments) as appropriated.

### 2.3. Cell Culture

HuH7, human hepatoma cells 7 clone 5 (passage 49), were obtained from Ceinge Biotecnologie Avanzate (Naples, Italy). Despite their tumoral nature, these cells possess a stable hepatic phenotype (response to insulin, production of cholesterol, lipids, and plasma proteins) and even when kept in culture for long-time do not de-differentiate nor accumulate epigenetic changes [33]. Cells were cultured (till passage 80) in Dulbecco Modified Eagle Medium (DMEM) (41965-039, GIBCO, Thermo Fisher Scientific, Waltham, MA, USA) supplemented with 10% FBS (10270, GIBCO), penicillin and streptomycin (15070-063, GIBCO) in a cell culture incubator at 37 °C and with 5% CO_2_. When indicated, Taurisolo (400 mg/L or 800 mg/L) or vehicle were added to the culture for 24, 48, or 72 h.

### 2.4. Animals for In Vivo Experiments

Wild-type male and female C57BL/6 mice (7 weeks old, postnatal day 49) were used in all experiments. All animals received human care and were placed in separate groups within standard mouse cages, ensuring their basic physiological and behavioral needs. Water was weighed daily, and fresh drinking water was prepared every two days. For the first 4 weeks, mice were fed regular chow (Mucedola, Settimo Milanese, Italy) starting at 5th week the animals were fed high fat diet (HFD) (TD06414, Mucedola, Italy) for an additional 4-weeks period. HFD was provided every two days and food intake was continuously monitored throughout the experiment. In addition, body weights were recorded before receiving the HFD and immediately before the sacrifice. Experiments were performed using a total of n. 6 mice receiving placebo and n. 6 mice receiving Taurisolo. Animal experimental protocols were approved by the Animal Research Committee of the University of Naples Federico II (Authorization n. 354/2019-PR). By the end of the experiment, no statistically significant difference in body weight, body weight gain, food or water consumed were found between Taurisolo and Placebo group.

### 2.5. Mitochondrial Staining

Mitochondria staining of cells and animal tissues was achieved by incubation with MitoTracker^®^ Red CMXRos (Thermo Fisher Scientific). A dye working solution was prepared by diluting a stock solution (10 μM in DMSO) in DMEM to yield a final concentration of 100 nM [34]. For staining of in vitro samples, HuH7 were rinsed twice in PBS before adding the dye. For staining of ex-vivo tissues, mice livers (soon after animal sacrifice) were washed in PBS by flushing PBS in the portal vein. Cells and tissues were incubated in the presence of the probe for 40 min in a cell incubator at 37 °C and 5% CO_2_. At the end of the incubation, cells and tissues were rinsed three times in DMEM and once in PBS, fixed in 4% formaldehyde and visualized under a IRIS fluorescent microscope (Logos Biosystem, Gyeonggi-do, Korea) or when indicate under confocal fluorescent microscope Zeiss LSM800 (Zeiss, Jena, Germany) equipped with an electronically switchable illumination and detection.

### 2.6. Metabolomic Analysis

Upon treatment, 2 × 10^6^ HuH7 cells were rinsed three times in PBS to be then homogenized in 1 mL of pre-chilled methanol/water 1:1 solution containing 10 nmol of internal standards. Samples were centrifuged at 10,000 g for 10 min at 4 °C [35]. The resulting supernatants were collected and transferred into new Eppendorf tubes and stored at −80 °C. Upon thawing, samples were dried using a speedvac to be then resuspended in 1 mL of acetonitrile and centrifuged again. The supernatant was used for injection. Analyses were performed in direct infusion by Fourier-transform Ion Cyclotron Resonance (FT-ICR) Mass Spectrometry, employing a Hamilton syringe (250 μL) at a flow rate of 2 μL/min [29,30,31,36]. Data were acquired on a SolariX XR 7T (Bruker Daltonics, Bremen, Germany). The instrument was tuned with a standard solution of sodium trifluoracetate. Mass Spectra were recorded in broadband mode in the range 100–1500 *m*/*z*, with an ion accumulation of 20 ms, with 32 scans using 2 million data points (2M). Nebulizing (N2) and drying gases (air) were set at 1 and 4 mL/min, respectively, with a drying temperature of 200 °C. Both positive and negative ESI ionizations were employed. Five replicates of each injection were carried out. The instrument was controlled by Bruker FTMS Control, MS spectra were elaborated with Compass Data Analysis version 4.2 (Bruker), tentative identification of compounds based on accurate MS measurements and isotopic fine distribution (ISF) was performed by Metaboscape 4.0 (Bruker). Metabolites signals were normalized using internal standards. Comparisons and differences were analyzed for statistical significance by two-way Anova test and Bonferroni post-test analysis. All graphs, bars or lines indicate means and standard errors of the mean (s.e.m.). TMAO blood and hepatic levels were measured as previously described [28]. When indicated fold changes in metabolite concentration measured by mass spectrometry were confirmed using commercially available enzymatic kit: for glucose determination (code 10505 from DIACRON Labs, Grosseto, Italy), for TG determination (code 10508 from DIACRON Labs), for cholesterol determination (code 10173 from DIACRON Labs).

### 2.7. 2-NBDG Glucose Uptake Assay on HuH7 Cells

HuH7 cells were plated (5 × 10^3^/well) in a black, clear bottom, 96-well microtiter plate (Perkin Elmer, Waltham, USA) in a final volume of 100 μL/well of culture medium. Once cells had reached 80–90% of confluence, culture medium was carefully removed and replaced with 100 μL of HBSS containing 100 μM 2-DG, 0.4 g/L BSA, and 1.3 mM CaCl_2_ (in the absence of any growth factors or FBS). Plates were incubated at 37 °C for 1 h. HBSS was then supplemented with insulin (1 nM, 10 nM or 100 nM), Taurisolo (400 mg/L or 800 mg/L) in the presence or in the absence of the PI3K inhibitor Ly294002 (10 μM). Plates were incubated for an additional period of 30 min. At the end of this second incubation, cell medium was replaced with HBSS containing 100 μM 2-DG, 0.4 g/L BSA, and 1.3 mM CaCl_2_ supplemented with 6 μM 2-NBDG [37]. Plates were incubated with the fluorescent probe for 45 min to be then washed twice in PBS. Uptake of 2-NDBG was measured in a Perkin Elmer Envision 2105 Multiplate reader (Perkin Elmer), using the inbuilt monochromator and the following parameters: λ excitation 471 nm, λ emission 529 nm, monochromator cut off 360 nm. After the measurement of 2-NDBG, cells were fixed in 3.7% PFA for 30 min to be then permeabilized in 0.1% Triton X-100 in PBS and stained with the nuclear dye DAPI (30 μM) [38]. This second fluorescence measurement correlate with the total number of cells in each well and was used for normalization. DAPI fluorescence was measured using the following parameters: λ excitation 351 nm, λ emission 450 nm. Data analysis for glucose uptake is reported as the ratio between intracellular 2-NDBG fluorescence and DAPI fluorescence ± s.e.m.

## 3. Results

### 3.1. Taurisolo Stimulates Glycogen Synthesis and Reduces Glycolysis in Huh7 Cells

As first, we investigated Taurisolo effect on Human Hepatoma HuH7 cells, a cell line that recapitulates most of the function of healthy hepatocyte [33]. Direct infusion FT-ICR mass spectrometry is endowed with ultra-high mass accuracy and resolution and allowed us to perform a rapid cellular metabolic profiling. We searched for intracellular metabolites modulated by treatment with the nutraceutical. We tested two different dosages of the nutraceutical (400 mg/L and 800 mg/L of Taurisolo, 72 h) to then compare their metabolome with that of vehicle-treated cells.

We first profiled metabolites representing energy source for the cell. HuH7 are highly demanding in terms of energy, and use glucose, amino acids and fatty acids (FA) as fuel or building blocks for several anabolic processes (lipogenesis, cholesterogenesis, and protein synthesis). Compared to cells treated with vehicle, the most statistically significant differences emerged from HuH7 cells treated with 800 mg/L of Taurisolo.

**Figure 1:**
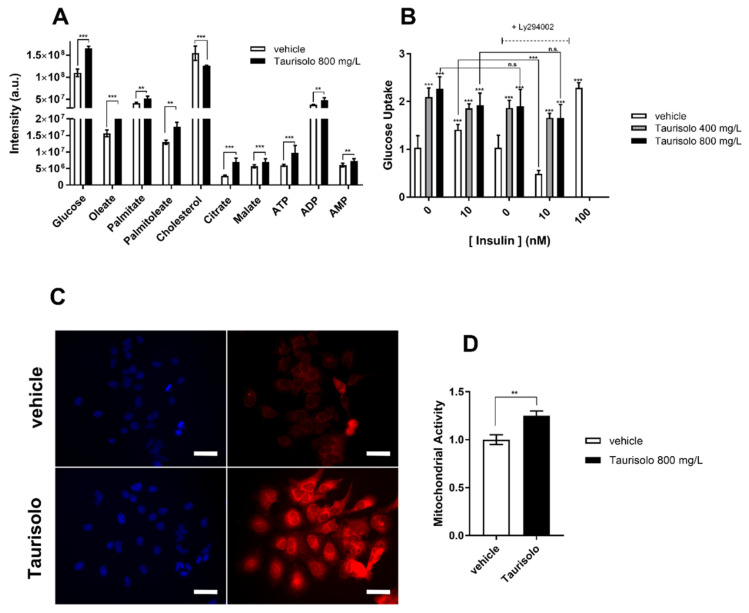
Taurisolo shifts HuH7 metabolism toward mitochondrial respiration. (**A**) Metabolomic profiling of HuH7 cells grown for 72 h in the presence of Taurisolo (800 mg/L) (black bars) or of vehicle (white bars) (see also Table 1). Each bar represents the mass spectrometry intensity of the indicated metabolites as measured by FT-ICR-MS. (**B**) Glucose uptake of HuH7 cells grown for 72 h in the presence of 400 mg/L (gray bars), 800 mg/L (black bars) Taurisolo, or of vehicle (white bars). When indicated 10 nM insulin and/or 10 μM LY294002 was added to the cell. (**C**,**D**) Fluorescent emission of MitoTracker CMX-ROS (red channel) visualized by fluorescence microscopy (**C**) or by spectrofluorimetric measurement (**D**) in HuH7 cells growing for 72 h in the presence of Taurisolo (800 mg/L) or of vehicle. Cell nuclei were counterstained with DAPI (blue channel). In D MitoTracker emission was normalized with DAPI to account for the cell number. Each bar represents normalized intensity of Mitotracker in HuH7 treated with 800 mg/L (black bars) Taurisolo, or with vehicle (white bars). In C scale bars correspond to 14 μm. (In (**A**,**B**,**D**) data are representative of *n* = 3 measurements, shown is mean ± s.e.m. Two-way ANOVA and Bonferroni post-test analysis were performed; *** = *p* < 0.001; ** = *p* < 0.01; ^n.s.^ = non-statistically different).

Intracellular levels of glucose and glucose-6-phosphate were increased by Taurisolo (fold increase of 1.50 ± 0.04 and 1.23 ± 0.05, respectively) (Figure 1A, Table 1). However, the two intermediates of glycolysis, namely fructose 1–6 diphosphate ad glyceraldehyde 3-phopshate, were not altered by the treatment, suggesting a fate for glucose other than glycolysis. The disaccharide maltose as well as UDP-glucose, both markers for glycogen synthesis, were both increased by Taurisolo (1.76 ± 0.14 and 1.48 ± 0.07) suggesting that, in cells treated with the nutraceutical, glucose is converted into glycogen.

### 3.2. Taurisolo Promotes Glucose Uptake in Cultured HuH7 Cells

Whether Taurisolo could indeed promote glucose uptake in HuH7 cells was then tested. The fluorescently labeled non-metabolizable glucose analogue 2-NBDG was used to directly monitor the uptake of the carbohydrate. Like unlabeled glucose, the probe is transported into the cytoplasm by the family of glucose transporters GLUTs. However, due to the absent hydroxyl group in position 2′, 2-NBDG cannot isomerize to Fructose-6-phosphate and accumulates into the cell without proceeding along the glycolytic pathway.

As shown in Figure 1B, 24 h treatment with either 400 mg/L or 800 mg/L of Taurisolo, promotes glucose uptake in HuH7 (2.0 ± 0.5 and 2.2 ± 0.5 over vehicle, respectively). Surprisingly, the amount of glucose uptake upon Taurisolo treatment is comparable to that promoted by 10 nM and 100 nM insulin, (1.3 ± 0.3 and 2.3 ± 0.5, respectively) suggesting that Taurisolo might stimulate GLUT transporters using an insulin-like mechanism (i.e., involving the AKT and PI3K kinases). To verify the PI3K-dependent Taurisolo effect on glucose uptake, the nutraceutical was administered to cells in the presence of the PI3K inhibitor Ly294002. As a consequence of the signaling cascade connecting insulin receptors to PI3K, glucose uptake following insulin stimulation was reduced by Ly294002. In contrast, Taurisolo activity was unchanged by Ly294002, suggesting a mechanism for the nutraceutical different from that used by insulin. Moreover, Taurisolo does not affect insulin activity, as the latter did not change in the presence of the nutraceutical. Finally, short time treatment (1 h) with either 400 mg/L or 800 mg/L Taurisolo did not induce glucose uptake (data not shown), arguing for Taurisolo mechanism of action as involving transcriptional regulation rather than activation of intracellular signaling.

### 3.3. Taurisolo Modulates Pentose Phosphate Pathway (PPP) in HuH7 Cells

Pentose phosphate pathway (PPP) is very active in liver cells. The first set of reactions of the pathway (the oxidative branch of PPP) allow the reduction of NADPH, an electron carrier needed for reductive biosynthesis reactions within the cell. NADPH is necessary to produce ribose-5-phosphate, as well as to maintain glutathione (GSH) in its reduced form. The first metabolite of the oxidative branch of PPP, 6-phospho gluconate (1.31 ± 0.04) is increased in Taurisolo-treated cells (Table 1). However, ribose-5-phosphate (0.79 ± 0.04) and ribulose -5-phosphate (0.65 ± 0.04) are reduced by Taurisolo suggesting a reduction of oxidative PPP in Taurisolo-treated cells. NADPH produced by PPP reduces oxidized glutathione produced in conditions of oxidative stress. As expected by a strong antioxidant like Taurisolo, GSH levels were increased in treated cells (1.24 ± 0.05). The stable antioxidant environment imposed by the nutraceutical is probably the reason behind downregulation of the oxidative branch of PPP.

The non-oxidative branch of PPP and sedoheptulose 7-phosphate (2.18 ± 0.08) and its byproducts sedoheptulose (2.21 ± 0.07), sedoheptulose 1,7-diphosphate (1.80 ± 0.08) were increased following exposure to Taurisolo (Table 1), that stimulates this alternative route producing glycolysis intermediates fructose-6-phosphate, glyceraldehyde-3-phosphate either for gluconeogenesis or for pyruvate production.

### 3.4. Taurisolo Induces Amino Acid Catabolism in HuH7 Cells

Apart from leucin (1.69 ± 0.01), intracellular levels of both ketogenic and glucogenic amino acids are either unaltered (e.g., phenylalanine and asparagine) or decreased by Taurisolo (Table 1). Histidine (0.69 ± 0.03), aspartate (0.75 ± 0.02), threonine (0.67 ± 0.02), tyrosine (0.82 ± 0.02), tryptophan (0.81 ± 0.02), glutamate (0.68 ± 0.02) were all reduced in cells treated with Taurisolo. Their decrease might be attributed either to their utilization for protein production or their conversion in glycolysis or Krebs cycle intermediates. Taurine (1.59 ± 0.05) (an antioxidant amino acid derivative of cysteine involved in cell protection from osmotic stress) is increased by Taurisolo. Interestingly, arginine is decreased (0.73 ± 0.03) probably converted into its derivative ornithine (1.33 ± 0.04) but not into citrulline that resulted non-statistically changed by Taurisolo. This is different from the data we obtained in endothelial cells where Taurisolo was found to stimulate arginine to citrulline conversion for NO production.

### 3.5. Taurisolo Stimulates Mitochondrial Activity in HuH7 Cells

In our metabolic profiling, citrate levels resulted increased by Taurisolo (1.46 ± 0.1 fold) (Figure 1 and Table 1), supporting the hypothesis of an increased acetyl-CoA production in mitochondria. In cells like hepatocytes, where cholesterogenesis occurs, citrate is rapidly exported out of the mitochondria and used as substrate to produce malonyl-CoA, necessary for biosynthesis of cholesterol. Intracellular cholesterol is however reduced by Taurisolo (0.78 ± 0.01) suggesting reduced cholesterogenesis (Figure 1 and Table 1). Similarly, synthesis of TG and plasma membrane lipids is hampered by Taurisolo as indicated by intracellular accumulation of free fatty acids (FA) palmitate (1.21 ± 0.04) and oleate (1.39 ± 0.06) and of two byproducts of plasma membrane lipids synthesis and degradation phosphoryl-choline (2.02 ± 0.07) and glycerol 1-palmitate (1.82 ± 0.04).

Overall, our metabolite profiling revealed that, in HuH7 cells, Taurisolo stimulates glycogen synthesis and allows the accumulation of citrate and free FA. Their increase argues for an increase in the mitochondrial activity as a consequence of augmented FA β-oxidation and Krebs Cycle. This hypothesis is supported by increased levels of malate (1.87 ± 0.01) and ATP (1.63 ± 0.06-fold) that we find in HuH7 cells treated with Taurisolo (Figure 1 and Table 1). These metabolites could increase in virtue of a higher mitochondrial respiration rate of Taurisolo-treated HuH7 cells.

To prove that the treatment with Taurisolo was indeed increasing mitochondrial respiration activity, we used the mitochondrial probe Mito Tracker CMX-ROS. The dye accumulates into cell organelles depending on their membrane potential. Since the membrane potential existing between the matrix and the mitochondrial inter-membrane space is an indication of mitochondrial activity, Mito Tracker CMX-ROS fluorescence intensity correlates with Oxidative Phosphorylation (OXPHOS). When analyzed by fluorimetry, HuH7 cells treated with Taurisolo showed an increased mitochondrial activity compared to cells treated with vehicle (1.20 ± 0.05, Figure 1C,D) confirming that Taurisolo stimulates mitochondrial activity. This is further supported by the reduction in the intracellular levels of creatine-phosphate (0.38 ± 0.02) indicating that the cells are not recycling ATP produced by glycolysis using the creatine cycle, due to the augmented biochemical activity of mitochondria.

Overall, in vitro experiments revealed that Taurisolo boosts mitochondrial activity in HuH7 cells. In contrast, several anabolic reactions occurring in the cytosol (glycogenolysis and PPP), as well as anabolic reactions occurring in the endoplasmic reticulum (membrane lipid biosynthesis and cholesterogenesis), are all diminished by Taurisolo. At least in vitro and as a consequence of the metabolic reprogramming, the nutraceutical was able to impose on hepatic cells, Taurisolo reduces three risk factors for NAFLD: it reduces cholesterogenesis and TG synthesis and improves hepatic glucose uptake.

### 3.6. Taurisolo Reduces Circulating TG Levels in C57BL/6 Mice Fed HFD

To confirm the hepatoprotective effect in vivo, Taurisolo [123 mg/kg/die, a dose equivalent to 800 mg/die (10 mg/kg/die in adults), the one suggested for human] or an equal amount of placebo, were administered to C57BL/6J mice. In order to induce the accumulation of cholesterol and triglycerides, mice from both groups were fed HFD. As previously shown, feeding mice HFD induces NAFLD and results in hepatic steatosis within 18 weeks. To monitor the ability of the nutraceutical to inhibit or delay the appearance of the two NAFLD risk factors the effect of Taurisolo was evaluated after 4 weeks of treatment. Upon treatment, mice were sacrificed and the metabolome of their serum and liver profiled by DI-FT-ICR-MS.

Since Taurisolo can reduce TMAO blood level in humans, we started measuring TMAO blood levels in mice to have a positive control of its activity. Taurisolo caused a marked decrease of TMAO (0.2 ± 0.1-fold difference between Taurisolo and placebo group, data not shown). While documenting the bioactivity of the tested nutraceutical, it confirms the ability of Taurisolo to reduce TMAO in this strain of mice. Oxidized vs Reduced Glutathione ratio was decreased by Taurisolo (0.7 ± 0.1-fold difference between Taurisolo and placebo group, data not shown) confirming, as shown by our previous results, the antioxidant activity of the nutraceutical. Blood TG levels were reduced by Taurisolo (111 ± 5 mg/dL, *n* = 6) as compared with placebo group (155 ± 4 mg/dL *n* = 6) confirming the ability of the nutraceutical to lower TG levels in mice fed HFD. Apart from that, mass spectrometry did not find any other statistically significant differences among the serum of Taurisolo and vehicle-treated animals. Enzymatic tests confirm mass spectrometry data and agreed in showing glucose and cholesterol were not affected by Taurisolo. Indeed blood glucose and cholesterol in Taurisolo group (198 ± 5 and 186 ± 4 mg/dL, respectively, *n* = 6,) were in a similar range of concentration compared to the placebo group (210 ± 4 and 178 ± 6 mg/dL, respectively *n* = 6), confirming a non-statistically significant variation.

### 3.7. Taurisolo Increases Glucose Levels in Hepatocytes of C57BL/6 Mice Fed HFD

**Figure 2:**
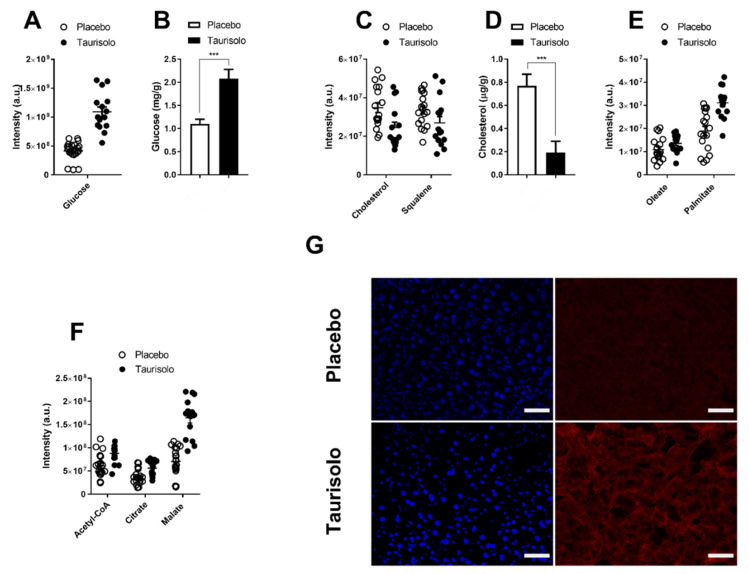
Taurisolo shifts mice liver metabolism toward mitochondrial respiration. (**A**–**F**) Metabolomic profiling of liver biopsies excised from mice fed High Fat Diet (HFD) and receiving Taurisolo (black dots) or Placebo (white dots) for 4 weeks (see also Table 2). Each dot represents the mass spectrometry intensity of the indicated metabolites as measured by FT-ICR-MS. In (**B**,**D**), mass spectrometry data for glucose and cholesterol were confirmed by measuring their intracellular concentration (using enzymatic reactions) in Taurisolo (black bars) or Placebo (gray bars) groups. (Data are representative of *n* = 6 triplicated measurements, shown is mean ± s.e.m. Two-way ANOVA and Bonferroni post-test analysis were performed; *** *p* < 0.001;). (**G**) The fluorescent emission of Mitotracker CMX-ROS (red channel) was here used to show the increase in mitochondrial membrane potential induced by Taurisolo in murine livers. Liver biopsies collected from mice treated with Taurisolo or placebo were incubated ex-vivo with Mitotracker CMX-ROS. UPPER PANEL: A faint fluorescence emission of the probe (red channel) is detectable in the hepatocytes of the placebo group. LOWER PANEL: Increased fluorescence emission of Mitotracker CMX-ROS in hepatocytes of the Taurisolo group. DAPI (blue channel) stains cell nuclei. In G, scale bars correspond to 50 μm.

Differently from serum, the metabolic profiling of mouse livers resulted statistically affected by Taurisolo. TMAO levels were drastically reduced in the liver of Taurisolo group compared with the Placebo one (0.3 ± 0.1) (Table 2). GSH (1.9 ± 0.3) and taurine (2.2 ± 0.3), were increased in the liver of Taurisolo group confirming the in vivo antioxidant potential of the nutraceutical (Figure 2 and Table 2). As measured in HuH7 cells, hepatic glucose levels were increased by Taurisolo (2.3 ±0.3). The glycolysis intermediates glucose 6-phosphate and fructose 1,6-diphosphate were not affected by the treatment, confirming also in vivo a fate for the up-taken glucose different from glycolysis. Glycogen intermediates UDP-glucose (1.7 ± 0.3) and maltose (1.6 ± 0.1) were increased confirming glucose conversion into glycogen. At variance with cultured cells, PPP resulted not influenced by Taurisolo (Table 2). These data likely reflect a difference between in vitro and in vivo settings, as well as, between tumoral (HuH7) and normal tissue.

### 3.8. Taurisolo Reduces Hepatic Cholesterol and TG Levels in C57BL/6 Mice Fed HFD

Hepatic FA palmitate (1.5 ± 0.1) palmitoleate (1.4 ± 0.1), oleate (1.4 ± 0.1), stearate (1.2 ± 0.1) and pentadecanoate (1.4 ± 0.1) were all increased in vivo by Taurisolo. Such increase indicates accelerated lipolysis as confirmed by the increase in the intermediate of lipid degradation, glycerol-1-phosphate (1.6 ± 0.1) (Figure 2 and Table 2). Like in vitro, the nutraceutical re-modulates cholesterogenesis in vivo, as shown by the decreased amount of cholesterol (0.7 ± 0.1), squalene (0.8 ± 0.1) and farnesyl (2,6)-di-phosphate (0.5 ± 0.2). Similarly, the amount of the two cholesterol derivatives cholic acid (0.5 ± 0.1) and taurocholic acid (0.5 ± 0.1), were decreased by Taurisolo probably due to the reduction of hepatic cholesterol and fat in the liver of the animals.

### 3.9. Taurisolo Stimulates Mitochondrial Activity in C57BL/6 Mice Fed HFD

Mitochondrial activity was increased in the liver of Taurisolo mice as shown by the increased amount of acetyl-CoA (1.4 ± 0.2), citrate (1.5 ± 0.2), malate (2.3 ± 0.3) and ATP (1.5 ± 0.3) (Figure 2 and Table 2). This was further confirmed by ex vivo staining of MitoTracker CMX-ROS. Soon after mice sacrifice, livers were excised, and blood was replenished with culture medium supplemented with the mitochondrial probe. The dye faintly stained the hepatocytes of Placebo mice (Figure 2G). In contrast, in the livers of Taurisolo mice, the fluorescence emitted by the probe resulted much more intense, confirming an increase in mitochondrial activity (Figure 2G).

Overall, the pool of statistically different metabolites in the Taurisolo group reveals that the nutraceutical induced a metabolic reprogramming of hepatocyte metabolism leading to increased mitochondrial activity. The nutraceutical stimulates glycogen synthesis, Krebs cycle, and OXPHOS activity, reducing membrane lipid biosynthesis and cholesterogenesis. As we measured in vitro, the consequence of the metabolic reprogramming imposed by Taurisolo reduces in vivo NAFLD risk factor markers of hepatic cholesterol and total TG, as well as improving hepatic glucose uptake.

## 4. Discussion

Despite the increasing number of nutraceuticals prescribed by physicians and their popularity as over-the-counter products, studies aimed at identifying their mechanism of action are far from being complete. Nutraceuticals are believed to generally act as antioxidants and supposed to reduce disease risk factors by lowering circulating and intracellular ROS levels [39]. However, an increasing number of reports suggest for them more complicated biochemical mechanisms [40].

The grape pomace extract of Aglianico grapes, in its nutraceutical formulation Taurisolo, contains high amounts of Resveratrol, Catechins, and Procyanidins [29]. Taurisolo has been initially formulated to act as a general antioxidant, useful to reduce excess of ROS produced during oxidative stress. However, in the years, Taurisolo has shown to affect many health parameters, with a mechanism that cannot be simply explained invoking its antioxidant activity. In human and in a murine model, Taurisolo reduces circulating blood levels of TMAO, a metabolite that ranks among the risk factor markers for cardiovascular disease [28]. Moreover, Taurisolo reduces blood-brain barrier leakage during an ischemic insult, reduces platelet-derived Thromboxane TxB2, and promotes Nitric Oxide production [29].

Here we provided evidence that Taurisolo (and very likely other grape pomace extracts) exerts hepatoprotective functions by reducing risk factor markers associated with NAFLD. The data supporting this hypothesis were collected in vitro in HuH7 cells treated for 72 h with 800 mg/L of Taurisolo and more importantly in vivo in C57BL/6 mice fed HFD and receiving Taurisolo (123 mg/Kg/die) for 4 weeks.

Both in vivo and in vitro, Taurisolo stimulates glucose uptake and the synthesis of glycogen, while reduces membrane lipid biosynthesis and cholesterogenesis in hepatocytes and reduces total circulating TG in blood and liver.

Even if often postulated, the molecular mechanism underpinning the health effect of nutraceuticals is still under debate. We are especially in an urge for new reports identifying and predicting the different effects exerted by nutraceuticals on different human cell types and the way these influence energy metabolism and metabolic choices of human organs. While science agrees in considering these gaps to be filled, the achievement of this milestone is hindered by the complex nature of nutraceuticals. The different biomolecules contained in Taurisolo, for example, all act synergistically and influence each other activity, ultimately affecting a biological scenario hard to decipher. The study of complex nutraceutical matrices can be however affronted by metabolomic approaches, extremely useful tools for probing any change in metabolic landscape accompanying nutraceutical treatments and providing invaluable insights in the mechanism of action of complex mixtures and phytocomplexes.

Here, we attempted a metabolic profiling of HuH7 cells as well as of serum and liver of mice treated with Taurisolo. In the experimental setting we used in vitro (72 h of treatment), Taurisolo led to a drastic change to the metabolic choices of cultured hepatocytes. In the experimental setting we used in vivo (male and female mice fed HFD and receiving Taurisolo) we found a statistically significant effect in the liver of the animals.

Metabolic analysis reveals that Taurisolo promotes OXPHOS and mitochondrial ATP production. Because of such increased mitochondrial activity, hepatic cells metabolism changes. Glucose uptake increases in a PI3K independent way. Catabolic reactions (necessary to fulfill mitochondrial requirements for intermediates), rather than anabolic ones, are favored. The rate of fatty acid β-oxidation, amino acid conversion to Krebs Cycle intermediates all increase, while the biosynthesis of cholesterol, bile acids, and plasma membrane lipids are disfavored. Such change in the cell metabolism helps to explain the hepatoprotective effect exerted in vivo by Taurisolo. This is in line with previous reports showing that pure resveratrol is able to stimulate OXPHOS in cells and tissues [41,42,43].

Several questions remain to be addressed to fully understand the molecular mechanism underpinning the healthy effect of Taurisolo. For example, new experiments will be needed to monitor Taurisolo effects on the gastrointestinal tract. Taurisolo contains Resveratrol, that has been shown to affect gut microbiota and favor the proliferation of specific commensal microbial strains in C57BL/6J mice [27]. Moreover, a recent study by Günther et al. [21] revealed that Resveratrol is able to inhibit gastrointestinal glucosidase activity and to induce glucose restriction and increased insulin sensitivity in C57BL-6J mice. The mechanism of action of Taurisolo, could indeed include remodeling of gut microbiota or inhibition of carbohydrate-hydrolyzing enzymes, amylase, and glucosidase, reducing the bioavailability of monosaccharides.

In vivo and in vitro data were coherent in many aspects. We found, however, an inhibitory effect exerted by Taurisolo on the PPP pathway in the in vitro culture but not in in vivo hepatocytes. One likely explanation for this discrepancy could be that tumoral hepatoma cells, like HuH7, manifest a Warburg effect for nucleotides. PPP is indeed necessary for producing nucleotides and deoxynucleotides used for cell replication and for gene expression. HuH7 cells could sustain their high rate of cell duplication boosting the PPP pathway. On the contrary, hepatocytes of adult healthy mice are probably residing in a more stationary phase of their cell cycle and thus require a less active PPP. Another possible reason could be that in vitro cultured cells normally rely on PPP to produce the amount of NADPH necessary to keep GSH in its reduced form. Taurisolo probably contributes to keeping GSH reduced, ultimately making PPP activity less necessary for HuH7 cells.

Another puzzling result is that Taurisolo strongly affects liver metabolome in vivo, leaving serum metabolites statistically unchanged. One explanation for this difference could be that the liver is a privileged target of chronic treatment with Taurisolo. At the same time, the duration chosen for the treatment was probably too short to measure statistically relevant changes in the serum of the animals. Moreover, after 4 weeks of HFD, only TG levels were over the average healthy range, while glucose and cholesterol levels of mice were not.

Notwithstanding, it remains that 4 weeks of treatment with Taurisolo were enough to reduce the hepatic levels of three risk factors for NAFLD. This is in line with previous reports showing pure Resveratrol [44] and nutraceuticals [17,45] able to improve liver function. Future experiments will be needed to reveal if the time window of treatments were indeed too short to measure an effect of Taurisolo in serum glucose and cholesterol concentration and if this can be achieved with longer treatment or higher doses of the nutraceutical.

## 5. Conclusions

The results here presented reveal some of the details behind the mechanism of action (at least in mice liver) of Taurisolo and support its eligibility as hepatoprotective nutraceutical for NAFLD prevention, ultimately adding an extra tile to the nutraceutical properties of Taurisolo.

## Figures and Tables

**Table 1:** Taurisolo reprograms the metabolism of HuH7 cells.

Metabolite	Taurisolo 400 mg/L	Taurisolo 800 mg/L	Metabolite	Taurisolo 400 mg/L	Taurisolo 800 mg/L
TMAO	0.52 ± 0.03 ***	0.43 ± 0.04 ***	Cholesterol	0.92 ± 0.03 ^n.s.^	0.78 ± 0.01 ***
Glucose	1.18 ± 0.03 *	1.50 ± 0.04 ***	Myristate	1.03 ± 0.02 ^n.s.^	1.06 ± 0.02 ^n.s.^
Glucose 6-P	1.01 ± 0.03 ^n.s.^	1.23 ± 0.05 *	Oleate	1.25 ± 0.04 **	1.39 ± 0.06 ***
Fructose-1,6-PP	0.94 ± 0.03 ^n.s.^	0.91 ± 0.03 ^n.s.^	Palmitate	1.07 ± 0.03 ^n.s.^	1.21 ± 0.05 **
Glyceraldehyde 3P	0.97 ± 0.03 ^n.s.^	0.88 ± 0.05 ^n.s.^	Palmitoleate	1.04 ± 0.03 ^n.s.^	1.37 ± 0.04 **
6-P-Gluconate	1.19 ± 0.07 ^n.s.^	1.31 ± 0.04 **	Phosphoryl-choline	1.26 ± 0.05 **	2.02 ± 0.07 ***
Ribulose-1,5-PP	0.89 ± 0.08 ^n.s.^	0.79 ± 0.04 ***	Leucin	1.29 ± 0.01 **	1.69 ± 0.01 ***
Ribose-5-P	0.87 ± 0.03 *	0.65 ± 0.04 ***	Tryptophan	0.62 ± 0.02 ***	0.81 ± 0.02 ***
Sedoheptulose	1.47 ± 0.03 **	2.21 ± 0.07 ***	Phenylalanine	0.86 ± 0.03 ^n.s.^	0.92 ± 0.06 ^n.s.^
Sedoheptulose-1,7-PP	1.30 ± 0.04 **	1.81 ± 0.09 ***	Tyrosine	0.81 ± 0.01 ***	0.82 ± 0.02 ***
Sedoheptulose-7-P	1.40 ± 0.03 **	2.18 ± 0.08 ***	Threonine	0.83 ± 0.03 ***	0.67 ± 0.03 ***
UDP-Glucose	1.67 ± 0.06 **	1.48 ± 0.07 **	Aspartate	0.84 ± 0.02 ***	0.75 ± 0.02 ***
Maltose	1.20 ± 0.07 *	1.76 ± 0.14 **	Glutamate	0.82 ± 0.02 ***	0.68 ± 0.02 ***
Malate	0.98 ± 0.03 ^n.s.^	1.87 ± 0.01 ***	Histidine	0.67 ± 0.05 ***	0.69 ± 0.07 ***
Citrate	1.29 ± 0.03 *	2.46 ± 0.14 ***	Arginine	0.45 ± 0.03 ***	0.73 ± 0.03 ***
ATP	1.01 ± 0.04 ^n.s.^	1.63 ± 0.06 ***	Ornithine	1.31 ± 0.06 ***	1.33 ± 0.04 ***
ADP	1.02 ± 0.02 ^n.s.^	1.26 ± 0.05 **	Asparagine	1.36 ± 0.06 ***	1.23 ± 0.04 **
AMP	0.96 ± 0.04 ^n.s.^	1.21 ± 0.05 **	Citrulline	0.94 ± 0.02 ^n.s.^	0.95 ± 0.02 ^n.s.^
Creatine-P	0.67 ± 0.03 ***	0.38 ± 0.02 ***	Taurine	1.19 ± 0.04 *	1.59 ± 0.05 ***
Glycerol 1-palmitate	1.00 ± 0.05 ^n.s.^	1.82 ± 0.04 **	GSH	1.19 ± 0.03 *	1.24 ± 0.05 **

Fold change (over vehicle) measured for the indicated metabolites in cells treated with 400 mg/L or 800 mg/L of Taurisolo for 72 h. Metabolites were extracted from HuH7 and analyzed by Mass Spectrometry as described in the methods section. (*n* = 3. Shown is mean ± s.e.m.) *** = *p* < 0.001; ** = *p* < 0.01; * = *p* < 0.05; ^n.s.^ non-statistically different from vehicle.

**Table 2:** Taurisolo reprograms metabolism of mice livers.

Metabolite	Taurisolo 800 mg/L	Metabolite	Taurisolo 800 mg/L
TMAO	0.3 ± 0.1 ***	Cholesterol	0.7 ± 0.1 **
Glucose	2.3 ± 0.3 ***	Squalene	0.8 ± 0.1 *
Glucose 6-P	1.3 ± 0.1 *	Farnesyl-2,6-PP	0.5 ± 0.2 **
Fructose-1,6-PP	1.1 ± 0.2 ^n.s.^	Linoleate	1.3 ± 0.1 **
6-P-Gluconate	1.5 ± 0.2 *	Stearate	1.2 ± 0.1 *
Ribulose-1,5-PP	1.2 ± 0.2 ^n.s.^	Oleate	1.4 ± 0.2 **
Ribose-5-P	1.1 ± 0.2 ^n.s.^	Palmitate	1.5 ± 0.1 **
Sedoheptulose	1.1 ± 0.2 ^n.s.^	Palmitoleate	1.4 ± 0.2 **
Sedoheptulose-1,7-PP	1.1 ± 0.3 ^n.s.^	Pentadecanoate	1.4 ± 0.1 **
Sedoheptulose-7-P	1.1 ± 0.1 ^n.s.^	Cholic acid	0.5 ± 0.1 ***
Acetyl-CoA	1.4 ± 0.2 *	Deoxycholic acid	0.6 ± 0.1 ***
UDP-Glucose	1.7 ± 0.3 **	Taurocholic acid	0.5 ± 0.1 ***
Maltose	1.6 ± 0.1 **	Glutamate	0.6 ± 0.1 ***
Malate	2.3 ± 0.3 ***	Glutamine	0.4 ± 0.1 ***
Citrate	1.5 ± 0.2 ***	Arginine	0.6 ± 0.2 **
Succinate	1.3 ± 0.1 **	Ornithine	1.3 ± 0.2 **
ATP	1.5 ± 0.3 **	Citrulline	1.0 ± 0.1 ^n.s.^
ADP	2.1 ± 0.3 ***	Aspartate	1.0 ± 0.2 ^n.s.^
AMP	1.3 ± 0.1 *	Taurine	2.2 ± 0.3 ***
Glycerol1-P	1.6 ± 0.1 **	GSH	1.9 ± 0.3 ***

Fold change (over PLACEBO) measured for the indicated metabolites in liver-fed HFD and receiving 123 mg/kg/die of Taurisolo. Metabolites were extracted from mice liver and analyzed by Mass Spectrometry as described in the methods section. 1 (*n* = 12. Shown is mean ± s.e.m.) *** = *p* < 0.001; ** = *p* < 0.01; * = *p* < 0.05; ^n.s.^ non-statistically different from PLACEBO.
